# Renal malignant perivascular epithelioid cell tumor: a case report and literature review

**DOI:** 10.3389/fonc.2026.1790155

**Published:** 2026-03-30

**Authors:** Shilong Li, Neng Zhang, Yongjin Yin

**Affiliations:** Department of Urology, Affiliated Hospital of Zunyi Medical University, Zunyi, China

**Keywords:** angiomyolipoma, malignant mesenchymal tumor, mTOR inhibitor, renal PEComa, tuberous sclerosis complex

## Abstract

**Background:**

Perivascular epithelioid cell tumors (PEComas) are rare mesenchymal neoplasms characterized by melanocytic and smooth muscle differentiation. Although the kidney is a relatively common site, malignant renal PEComa is exceedingly rare, particularly in patients with tuberous sclerosis complex (TSC). Overlapping radiologic features between angiomyolipoma and malignant PEComa often result in diagnostic challenges.

**Case presentation:**

We report an 18-year-old female who presented with right flank pain. Contrast-enhanced CT revealed a large heterogeneous mass in the right kidney (127 × 81 mm) with intratumoral hemorrhage, initially interpreted as angiomyolipoma associated with TSC. Physical examination and family history were suggestive of TSC, and genetic testing confirmed a pathogenic TSC2 frameshift mutation. The patient underwent open right partial nephrectomy. Histopathology demonstrated a malignant PEComa composed of epithelioid cells with focal coagulative necrosis. Immunohistochemistry showed diffuse positivity for Melan-A and smooth muscle actin, focal HMB-45 expression, and a Ki-67 index of approximately 5%. The patient recovered postoperatively; however, CT imaging at one-month follow-up revealed a suspected enhancing mass in the lateral mid-portion of the right kidney, requiring continued close surveillance. Based on the suspected enhancing mass identified at the one-month postoperative CT follow-up and the confirmed malignant potential, the patient has been advised to initiate adjuvant mTOR inhibitor therapy (Everolimus). A rigorous 3-month interval follow-up protocol has been established to evaluate treatment efficacy and prognosis.

**Conclusion:**

This case highlights a rare occurrence of malignant renal PEComa in a young patient with genetically confirmed TSC. Large tumor size, necrosis, and mitotic activity are important indicators of malignant potential. In young patients with TSC and atypical renal masses, malignant PEComa should be considered in the differential diagnosis. Multidisciplinary evaluation, genetic testing, and awareness of emerging mTOR-targeted therapies are essential for optimal management.

## Introduction

Perivascular epithelioid cell tumors (PEComas) are extremely rare mesenchymal neoplasms composed of epithelioid cells with both melanocytic and smooth muscle features (1, 2). Renal involvement is common among visceral PEComas (3), but truly malignant renal PEComas are extraordinarily uncommon. On imaging, renal PEComas often mimic typical angiomyolipoma (AML) or renal cell carcinoma (4), making preoperative diagnosis difficult. A subset of renal PEComas is associated with tuberous sclerosis complex (TSC), an autosomal dominant disorder caused by pathogenic variants in TSC1 or TSC2 (5). Loss of the hamartin-tuberin complex leads to uncontrolled activation of the mTOR pathway, driving tumorigenesis. Although most PEComas arise sporadically, patients with TSC have a substantially higher incidence of PEComa and often develop multiple or bilateral renal lesions at a younger age (5). Because malignant renal PEComa is so rare and treatment guidelines are limited, detailed case reports and literature reviews are important to inform diagnosis and therapy. We describe an 18-year-old female with TSC-associated malignant renal PEComa and review relevant literature, focusing on the TSC association, prognostic factors, and advances in treatment.

## Case presentation

An 18-year-old female presented to our department with a chief complaint of right flank pain persisting for over 20 days. Prior to admission, the patient presented to the Dermatology outpatient clinic and received a preliminary diagnosis of Tuberous Sclerosis Complex (TSC).

### Patient presentation and family history

**Figure 1:**
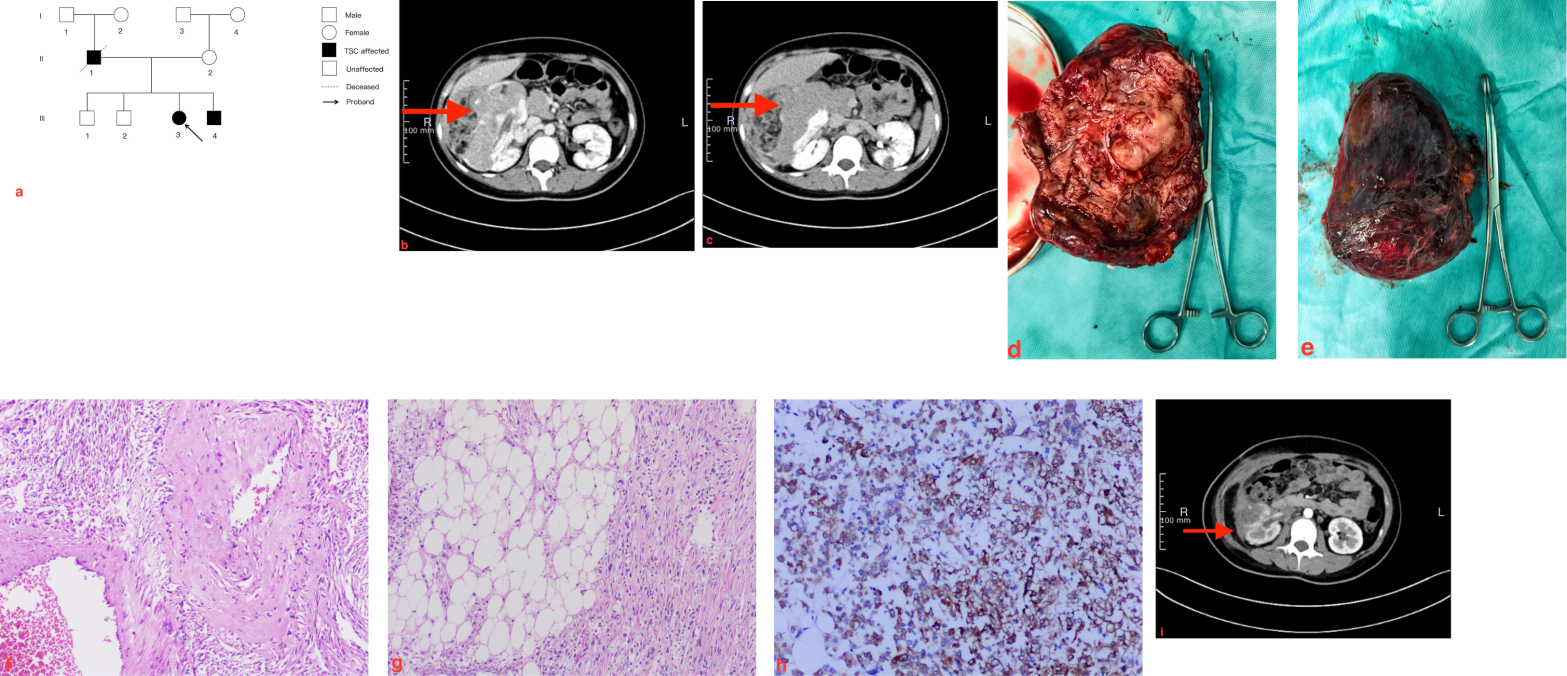
Genetic pedigree chart labeled **(a)** shows affected and unaffected individuals in three generations, highlighting the proband with an arrow. Labeled **(b, c)**, two abdominal CT scans display a solid renal mass marked by red arrows. Images **(d, e)** respectively show the front and back sides of the large, irregularly shaped kidney tumor that was surgically removed at the clamp site beside the surgical towel. Images **(f, g)** are microscopic photographs of tumor tissue stained with hematoxylin and eosin, demonstrating abnormal spindle and adiposecells. Image **(h)** is a microscopic photo showing immunohistochemical staining of tumor cells. Image **(i)** is another abdominal CT scan after one month of the operation with a red arrow indicating a smaller renal lesion.

Her past medical history was notable for the onset of multiple papules on the nasolabial folds, chin, cheeks, and forehead approximately 10 years prior, which had remained untreated. Family history was significant for similar facial cutaneous manifestations in both her father and younger brother; notably, her father was suspected to have succumbed to complications of a related underlying pathology, and her brother had a documented history of epilepsy. Neurologically, the patient had no history of seizures or cognitive impairment. Although an EEG(Electroencephalogram) was not performed during hospitalization, cranial MRI (magnetic resonance imaging) showed no evidence of cortical tubers or subependymal giant cell astrocytomas (SEGA), aligning with her asymptomatic neurological status. Given that tuberous sclerosis complex (TSC) is an autosomal dominant disorder, the patient has been subsequently confirmed to have TSC type 2 and is considered the “proband” of this familial hereditary condition (**Figure 1A**).

Physical examination revealed multiple facial angiofibromas (distributed in a butterfly pattern across the nose and cheeks) and two hypomelanotic macules (ash-leaf spots) on the patient’s trunk, which are classic cutaneous markers of TSC. Abdominal palpation identified a firm mass in the right abdomen, accompanied by tenderness at the right upper and middle ureteral points and positive percussion tenderness over the right renal area. Neurological signs are absent. In view of the potential for TSC-associated complications, including retinal gliomas and neurological deficits such as epilepsy, the patient was evaluated via fundoscopy and brain MRI. The results of these investigations were unremarkable. Laboratory investigations indicated mild anemia (hemoglobin 99.0 g/L), while urinalysis, liver and kidney function tests, and tumor markers were within normal limits.

### Radiological evaluation

Computed tomography (CT) of the kidneys (**Figures 1B, C**) demonstrated a markedly enlarged and deformed right kidney occupied by a large, irregular, heterogeneous mass measuring approximately 127×81 mm. The lesion contained areas of fat attenuation and hemorrhage, showing progressive uneven enhancement. The left kidney exhibited multiple focal low-density lesions and a nodule measuring 17×16 mm. Additionally, chest CT revealed multiple small pulmonary nodules with ground-glass opacity; however, the patient was clinically asymptomatic, reporting no cough, shortness of breath, or dyspnea. The liver showed suspected multiple cysts, and the remainder of the abdominal viscera appeared unremarkable. Based on the comprehensive findings, the preoperative diagnosis was established as Angiomyolipoma of the right kidney associated with Tuberous Sclerosis Complex.

### Surgical intervention and histopathology

Given the presence of intratumoral hemorrhage and the patient’s young age, the patient subsequently underwent a right open partial nephrectomy with ureteral stent placement. Intraoperatively, a giant tumor measuring 12×10×8 cm was identified adhering to the renal pelvis and surrounding tissues; the mass was completely resected with distinct margins (**Figures 1D, E**). Gross pathological examination revealed a solid, grayish white to grayish-yellow nodular mass with focal hemorrhagic areas, confined within the renal capsule. Postoperative CT imaging indicated status post right partial nephrectomy with evidence of post-surgical hematoma and seroma in the operative bed. The right kidney showed signs of reduced renal perfusion. After treatment consisting of sufficient surgical field drainage, anti-infective agents, and intravenous fluids, the patient recovered well. Hepatic and renal function, along with electrolytes, normalized. Postoperative Hb levels were stable compared to pre-operation, but anemia remained, necessitating long-term convalescence. The patient was discharged smoothly on Postoperative Day 10 without immediate adjuvant therapy (mTOR inhibitors) or chemoradiotherapy.

**Figure 2:**
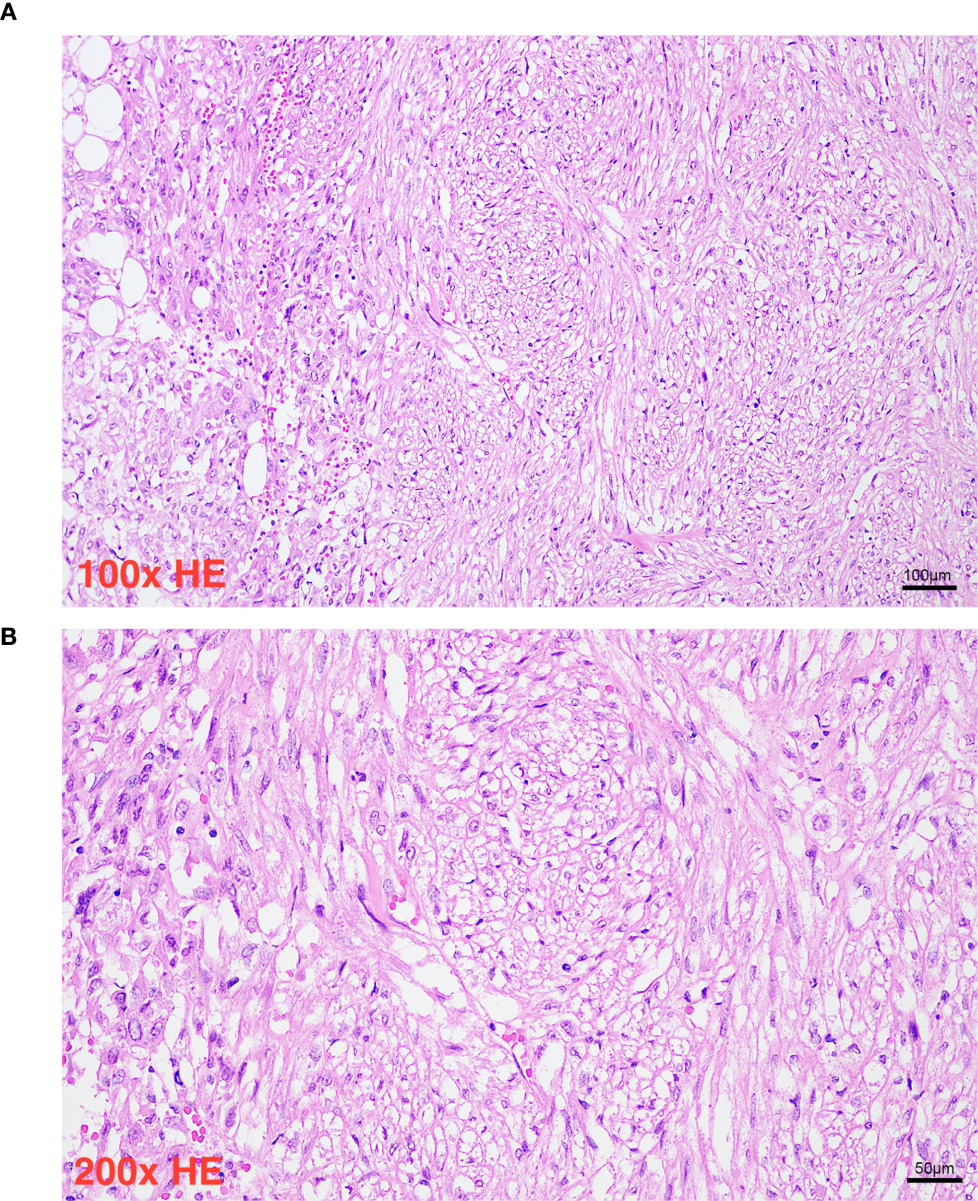
Panel **(A)** shows a histology slide at one hundred times magnification stained with hematoxylin and eosin, demonstrating spindle-shaped cells and regions with clear cytoplasm. Panel **(B)** presents a similar tissue section at two hundred times magnification, providing greater cellular detail with more prominent nuclei and vascular structures. Both images include scale bars indicating one hundred micrometers and fifty micrometers, respectively.

**Figure 3:**
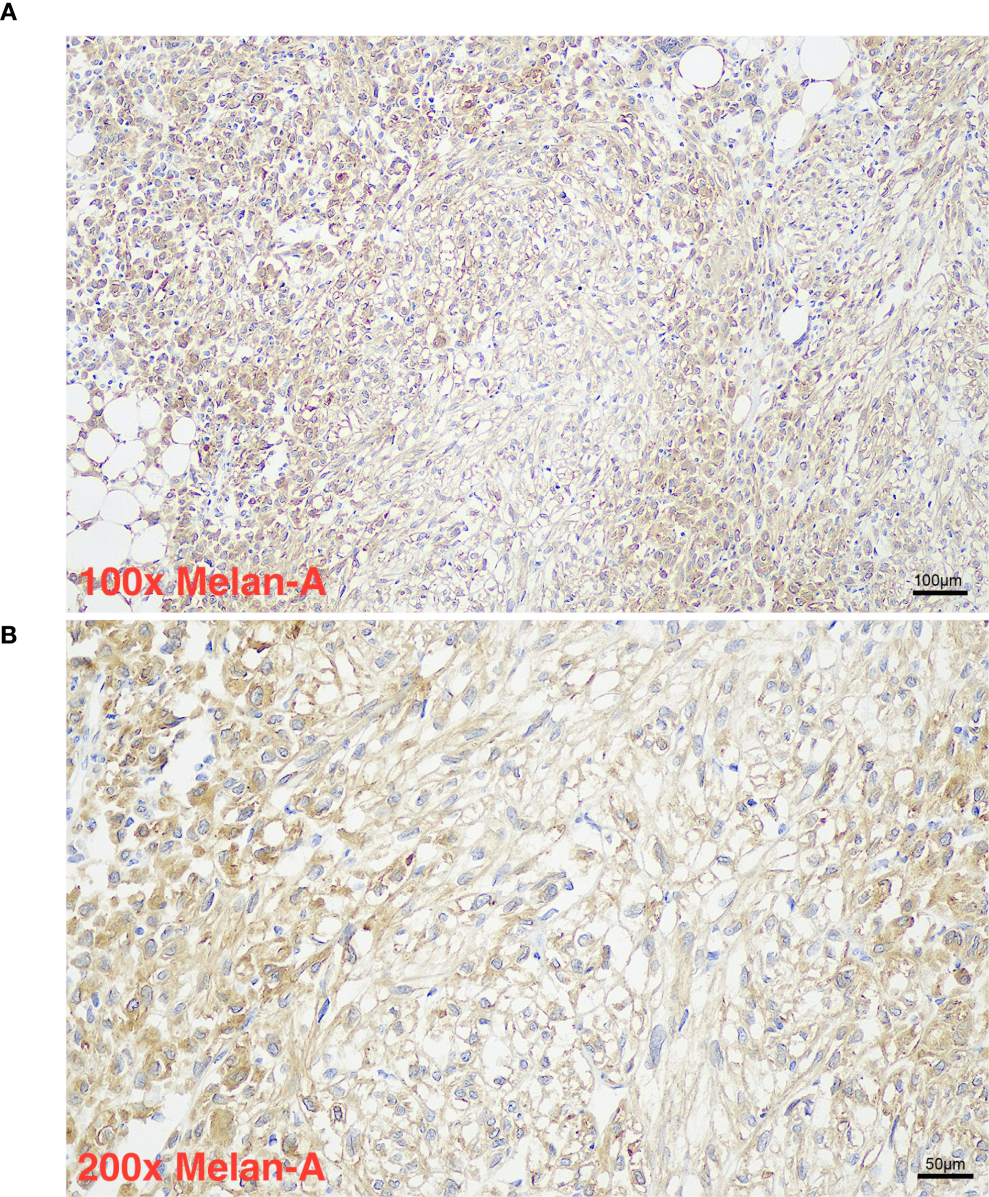
Panel **(A)** shows a microscopic image at 100 times magnification with Melan-A immunohistochemical staining, highlighting brown-stained cells within a tissue section. Panel **(B)** presents a closer view at 200 times magnification, emphasizing individual cell morphology and brown Melan-A positive staining. Scale bars indicate 100 micrometers in panel **(A)** and 50 micrometers in panel **(B)**.

**Figure 4:**
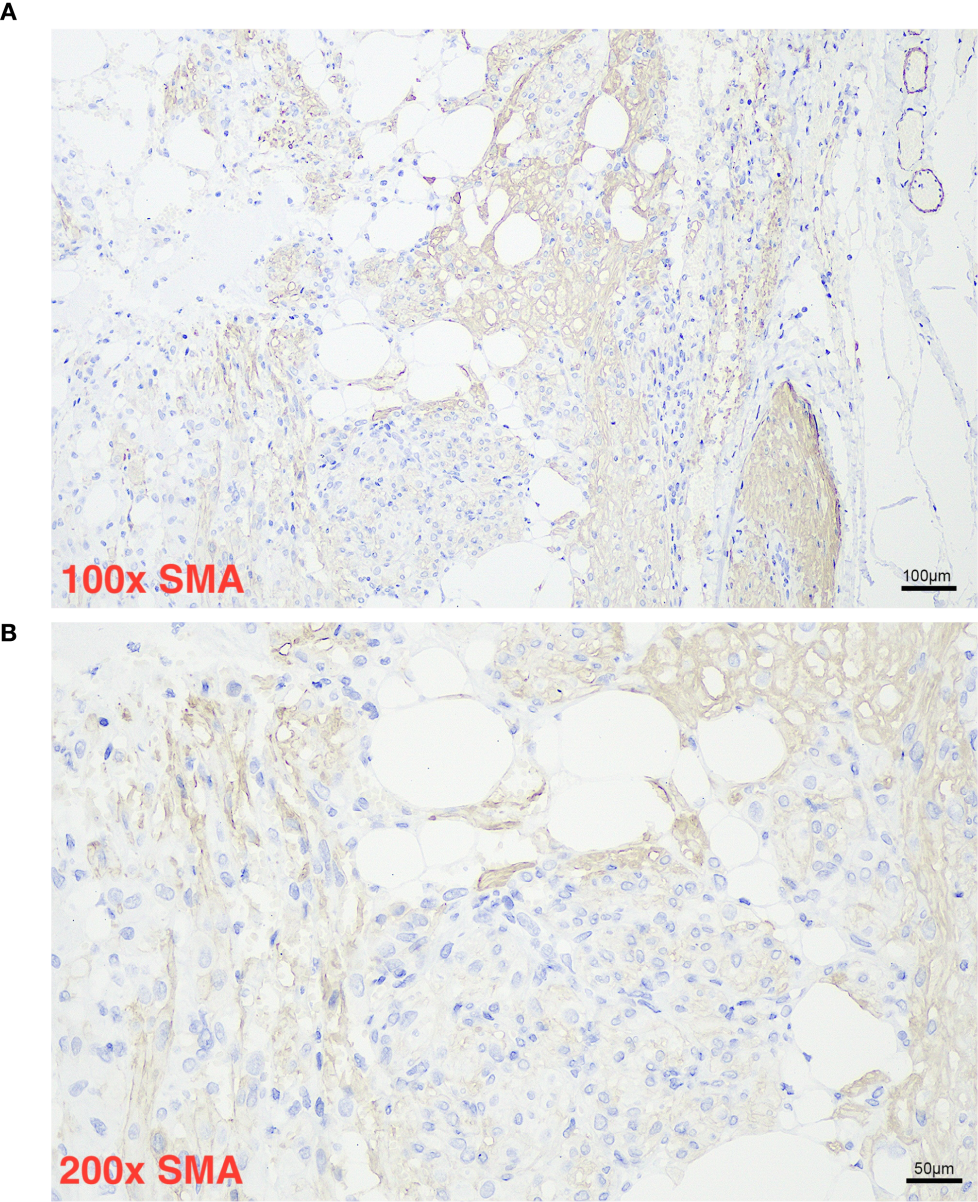
Panel **(A)** shows a microscopic section of tissue stained for smooth muscle actin (SMA) atone hundred times magnification, with brown staining highlighting SMA-positive cells among white adipocytes and blue nuclei. Panel **(B)** presents a higher magnification of the same tissue at two hundred times, again demonstrating SMA positive regions, white adipocytes, and blue-stained nuclei, with the scale bars indicating one hundred micrometers and fifty micrometers respectively.

**Figure 5:**
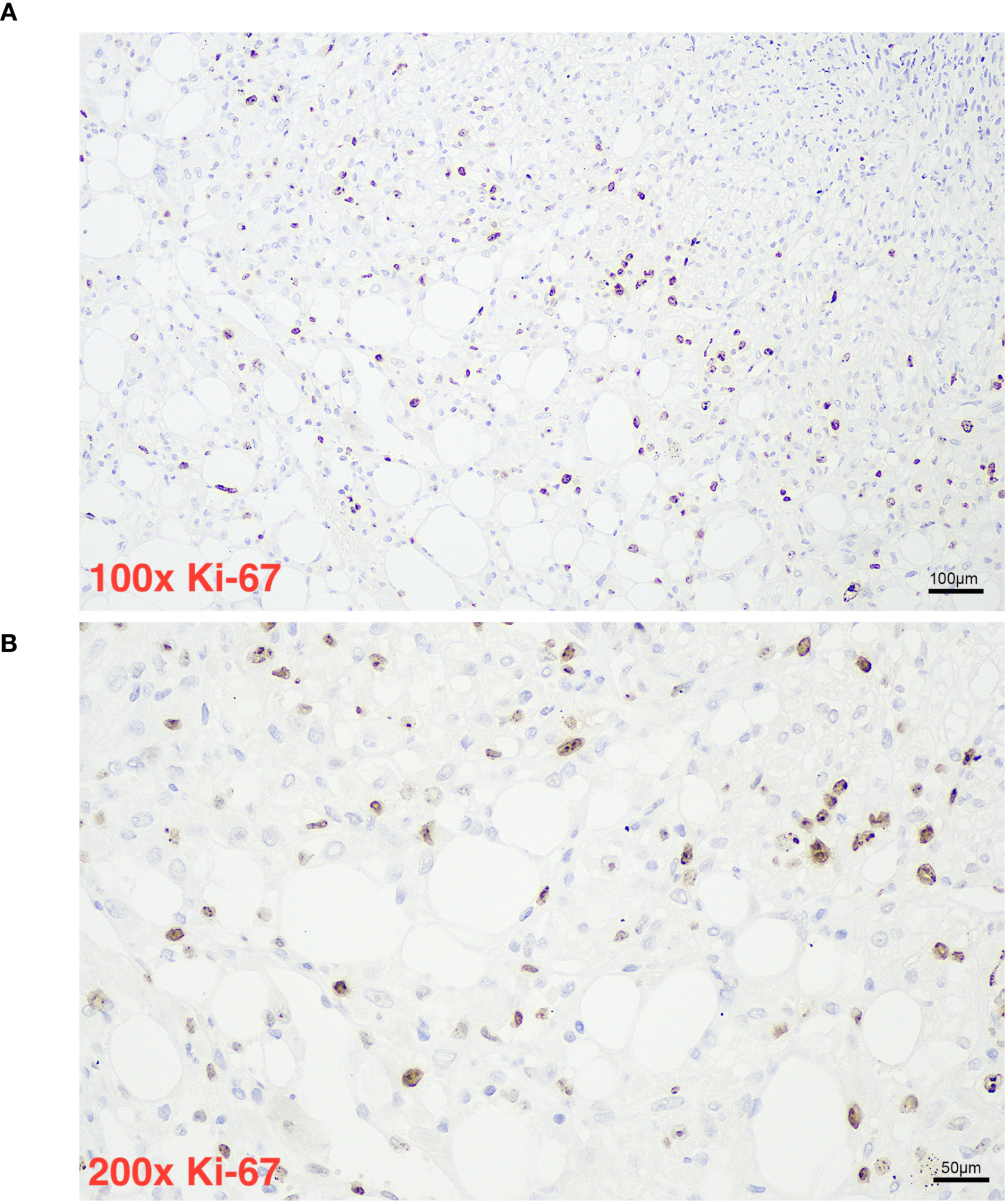
Panel **(A)** shows a histological slide of tissue stained for Ki-67 at 100 times magnification with numerous brown-stained nuclei, indicating proliferating cells, surrounded by pale cytoplasmic spaces. Panel **(B)** presents a higher magnification, 200 times, of a similar area, illustrating increased detail of Ki-67 positive nuclei and cellular morphology with a scale bar showing 50 micrometers.

Histopathological evaluation (**Figures 1F, G**, **2A, B**) demonstrated a triphasic pattern comprising mature adipose tissue, thick-walled blood vessels, and a proliferation of spindle and epithelioid cells. The tumor cells possessed abundant eosinophilic cytoplasm with focal rhabdoid features and significant nuclear atypia. Coagulative necrosis was present, and the mitotic rate exceeded 1 per 50 high-power fields (HPF). Immunohistochemical staining (**Figure 1H**) showed positivity for Melan-A (**Figures 3A, B**), SMA (**Figures 4A, B**), and Vimentin, and focal positivity for HMB45, while CK, Desmin, and PAX-8 were negative; the Ki-67 (**Figures 5A, B**) labeling index was approximately 5%.

### Result of the gene detection

Whole-exome sequencing (WES) was performed using genomic DNA extracted from peripheral blood. Sequencing achieved high coverage across the targeted regions (average on-target depth 281×; >20× coverage 99.65%). A single pathogenic variant relevant to the patient’s phenotype was identified.

Genetic detection shows that A heterozygous frameshift mutation in TSC2 was detected: NM_000548.5: c.1016_1023dup (p.Ile342SerfsTer24).

This 8-bp duplication causes a frameshift and introduces a premature termination codon, predicted to result in truncated tuberin or nonsense-mediated mRNA decay. As loss-of-function is a well-established pathogenic mechanism for *TSC2*, this variant meets ACMG (American College of Medical Genetics and Genomics) criteria for pathogenicity (PVS1, PM2_supporting, PP4). The variant is novel and absent from major population databases.

The TSC2 mutation is associated with Tuberous Sclerosis Complex type 2 (autosomal dominant) and may also contribute to renal angiomyolipoma formation and other TSC-related manifestation. The finding is consistent with the patient’s renal presentation.

### Postoperative follow-up and treatment transition

Based on the tumor size (>5 cm), presence of necrosis, and high mitotic activity, the final diagnosis was malignant perivascular epithelioid cell tumor (PEComa) of the right kidney. A strict follow-up protocol was initiated to monitor for recurrence or metastasis given the malignant potential and genetic background. At the one-month postoperative follow-up, the patient was clinically stable; however, contrast-enhanced CT revealed a new, suspected enhancing mass in the lateral aspect of the operative bed (**Figure 1I**). Given this finding and the inherent high risk of recurrence associated with the original tumor’s size and necrosis, the treatment team recommended targeted therapy with Everolimus. Due to economic considerations, the patient is currently processing medical insurance applications and is scheduled to begin medication shortly. The follow-up plan has been finalized to include re-evaluations every 3 months, focusing on imaging of the kidneys, chest CT, and monitoring of mTOR inhibitor-related toxicities.

## Discussion

### Epidemiology and diagnostic challenges

PEComas are extremely rare, and exact incidence data are lacking. They occur predominantly in females and across a wide age range, whereas typical sporadic renal AMLs usually present around age 50 (6). An 18-year-old with malignant renal PEComa is extraordinarily unusual. Clinically, most patients have no specific symptoms, and lesions are often discovered incidentally or during workup for other conditions. Renal PEComas lack distinctive imaging features. On CT or MRI, they frequently appear as isodense or hypodense masses with heterogeneous enhancement (7). Studies report that preoperative imaging correctly identifies PEComa in almost none of cases, and these tumors are often misdiagnosed as renal cell carcinoma (8–10). Thus, definitive diagnosis requires histopathology with immunohistochemistry, demonstrating dual expression of melanocytic markers (HMB-45, Melan-A) and smooth muscle markers. Grossly, PEComas usually lack a true capsule and often show hemorrhage or necrosis. Microscopically, they are composed of epithelioid cells arranged radially around blood vessels; thick-walled vasculature is characteristic. No serum or biochemical markers are specific for PEComa, so diagnosis typically necessitates multidisciplinary evaluation.

### Association with tuberous sclerosis complex

Tuberous sclerosis complex (TSC) is an autosomal dominant disorder caused by pathogenic variants in *TSC1* (encoding hamartin) on chromosome 9q34 or *TSC2* (encoding tuberin) on 16p13.3 (5). Hamartin and tuberin form a heterodimeric complex that inhibits mTOR signaling (5, 11). Loss-of-function mutations in *TSC1/2* disable this inhibition, leading to unregulated mTOR activation and tumor formation (5, 11). Chromosomal analyses of PEComa tissues often reveal loss of *TSC2*, underscoring mTOR pathway activation in these tumors. Approximately 60–80% of patients with TSC develop renal AML or PEComa lesions, whereas most PEComa patients (80–90%) do not have overt TSC manifestations (12–14).

Typical clinical features of TSC include hypomelanotic macules (ash-leaf spots), facial angiofibroma, cortical tubers, epilepsy, and renal angiomyolipoma or cysts (5). TSC-related renal AMLs tend to present at a younger age (median <20 years) and are more often bilateral and multifocal (>80% of cases) with lesions usually ≤3 cm (15). In our patient, the early age and bilateral renal hamartomas with a *TSC2* mutation are consistent with TSC-related renal AMLs, although malignant transformation is very rare. Some authors have described a “sclerosing PEComa” variant, in which extensive hyalinization is seen (6); this subtype may also be related to TSC and has distinct immunogenetic features (16). In summary, *TSC1/2* mutations that deregulate mTOR signaling mechanistically link TSC and PEComa pathogenesis.

### Pathological criteria and prognostic factors

Folpe and colleagues proposed criteria for assessing PEComa malignancy based on pathological features (17). Tumors are considered malignant if they exhibit two or more of the following adverse features:

- Tumor size >5 cm.
- Infiltrative growth pattern.
- High-grade nuclear atypia.
- Presence of necrosis.
- Mitotic rate ≥1 per 50 high-power fields.
- Vascular or lymphatic invasion.

If only one adverse feature is present, the lesion is of uncertain malignant potential, and if none are present it is considered benign. In our case, the tumor exceeded 5 cm, had focal necrosis, and mitoses (≥1/50 HPF), fulfilling multiple criteria for malignancy.

Ki-67 proliferation index provides additional prognostic information: tumors with high Ki-67 (often >10–25%) tend to be more aggressive. Our patient’s Ki-67 was relatively low (5%), but the large size and necrosis justified a malignant classification. TFE3 gene rearrangement defines another molecular subset of PEComa, typically seen in younger patients with strong TFE3 immunostaining alongside melanocytic markers (18); these were initially thought mutually exclusive with TSC mutations, but recent data show they can coincide. In practice, integrating Folpe criteria with proliferation and molecular markers allows a more comprehensive risk assessment.

### Advances in mTOR inhibitors

Given the role of mTOR activation in TSC-associated tumors, mTOR inhibitors have become key targeted agents for PEComa and AML. Clinically, everolimus (an oral mTOR inhibitor) is routinely used in TSC patients with renal AML and has shown significant tumor shrinkage and delayed progression (19). A pivotal multicenter trial (EXIST-2) demonstrated that everolimus markedly reduced AML volume and lowered the risk of bleeding or need for surgery (20). These benefits have been confirmed in Chinese TSC populations as well (21). Sirolimus (rapamycin) and its analogs have similarly been applied in malignant PEComa cases, with reports of partial responses or disease stabilization (22, 23).

Notably, in November 2021 the U.S. Food and Drug Administration approved albumin-bound sirolimus nanoparticles (nab-sirolimus, trade name Fyarro) for treatment of adult patients with locally advanced unresectable or metastatic malignant PEComa. This approval was based on the AMPECT trial (24), which showed an objective response rate of 39% (1 complete and 11 partial responses) in 31 patients, with most responses lasting ≥12–24 months. Common adverse effects of nab-sirolimus included mucositis (stomatitis), rash, fatigue, infections, nausea, edema, and weight loss; ≥30% of patients experienced stomatitis, fatigue, or rash. Everolimus and sirolimus have similar toxicity profiles, including mucosal ulcerations, hyperglycemia, infections, edema, and pneumonitis. Overall, for locally advanced or metastatic malignant PEComa, mTOR inhibitors are now the systemic treatment of choice, especially in tumors with *TSC1/2* mutations or evidence of mTOR pathway activation.

### Comparison with reported cases

Previous reports (25–27) indicate that malignant renal PEComa (epithelioid AML) typically occurs in young to middle-aged adults of both sexes, with most cases being sporadic although some have TSC. Patients often present with flank pain, hematuria, or an abdominal mass. Imaging usually reveals a large solid intrarenal mass lacking obvious fat, with heterogeneous enhancement, leading to frequent preoperative misdiagnosis as renal cell carcinoma. Histopathologically, these tumors are predominantly composed of epithelioid cells with marked atypia, active mitoses, and necrosis. Immunohistochemistry shows strong HMB-45 and Melan-A expression, variable SMA positivity, and negativity for epithelial and neural markers, fitting the PEComa profile.

Large case series (27) (Brimo, Nese, et al.) and subsequent cohorts have identified poor prognostic factors: tumor diameter >7 cm, high proportion of epithelioid cells, mitotic rate ≥2/10 HPF, presence of necrosis, extrarenal extension or vascular invasion, and elevated Ki-67. Approximately 25–50% of patients develop distant metastases on follow-up, and roughly one-third die of disease, underscoring the malignant potential of these lesions. These data support the need for aggressive surgical resection and long-term surveillance.

The rarity and heterogeneity of malignant renal PEComa are further highlighted when comparing our case to the most recent report by Li et al. (2024) (28). While both cases involve young female patients, the biological behavior of the tumors differed significantly. The 28-year-old patient described by Li et al. (2024) presented with a relatively small, localized lesion (~12 mm). In contrast, our 18-year-old patient presented with a massive 12.7 cm tumor, which is among the largest reported in adolescent TSC patients.

More importantly, the timeline of progression varies drastically across literature. In the 2012 case by Li et al. (26), metastasis occurred 7 years post-resection, whereas our patient exhibited a suspected enhancing mass only one month after surgery. This comparison underscores two critical points:

1. Risk Stratification: The combination of younger age, TSC2 germline mutation, and massive tumor size (as seen in our case) may serve as a red flag for ‘hyper-aggressive’ PEComa phenotypes that require much earlier intervention than sporadic cases.
2. Surveillance and Intervention: While Li et al. (2024) suggest ‘long-term follow-up’, our case demonstrates that for high-risk individuals, the ‘long-term’ approach must be replaced by high-frequency early surveillance (e.g., 1-month and 3-month intervals) and the proactive use of mTOR inhibitors like Everolimus to preempt potential recurrence.”

Compared to sporadic cases, the diagnostic challenge in our 18-year-old patient was twofold. First, the peak incidence of sporadic renal PEComa is typically around age 50, making its occurrence in late adolescence extraordinarily rare. Second, in the context of TSC, clinicians often expect benign, slow-growing angiomyolipomas (AMLs). The literature suggests that preoperative imaging identifies PEComa in almost zero percent of cases, often misdiagnosing them as RCC. Our case underscores that in young TSC patients, the ‘benign assumption’ must be challenged when a mass exhibits atypical features like rapid growth or internal necrosis, even if it mimics a fat-poor AML.

The optimal management of localized malignant PEComa remains controversial. While surgical resection is the mainstay, the high rate of metastasis (up to 50%) reported in literature warrants consideration of adjuvant therapy. In our case, the decision-making process evolved dynamically. Initially, a ‘wait-and-see’ approach was adopted post-R0 resection. However, the discovery of a suspected enhancing mass at the one-month follow-up CT—a much earlier presentation of potential recurrence than typical reports—prompted a multidisciplinary re-evaluation. We have now recommended Everolimus (10 mg/day), a choice supported by the EXIST-2 trial and its proven efficacy in TSC-related renal lesions. This pivot emphasizes that for high-risk PEComas (size >5 cm, necrosis), the window for adjuvant intervention may be significantly narrower than previously thought.

Our case highlights a potential discrepancy between molecular markers and clinical behavior. Despite a relatively low Ki-67 (5%), the patient’s early imaging findings suggest a high-grade biology, validating the Folpe criteria’s emphasis on tumor size and necrosis. Furthermore, this case brings to light the socioeconomic barriers to optimal care. While mTOR inhibitors like Everolimus or nab-sirolimus are the ‘gold standard’ for malignant PEComa, their high cost necessitates a delay in treatment while the patient secures medical insurance coverage. This real-world constraint, coupled with a strict 3-month follow-up protocol, represents a pragmatic approach to managing rare, aggressive malignancies in developing healthcare settings.

### Future directions

Given the rarity of PEComa, multicenter collaborations are needed to establish large patient databases and conduct retrospective analyses to elucidate its pathogenesis and clinical behavior. Emphasis should be placed on molecular subtyping (for example, distinguishing *TSC1/2*-mutant vs. TFE3-rearranged PEComas, as in our case) to guide individualized therapy. Although mTOR inhibitors have shown promise, resistance invariably develops. Future studies could explore combination strategies, such as pairing mTOR inhibitors with other targeted therapies or immunotherapies. For instance, there are reports of combining everolimus with angiogenesis inhibitors or immune checkpoint inhibitors to achieve more durable responses (29). Dual inhibition of PI3K/AKT or MET pathways alongside mTOR blockade may also help overcome resistance. On the basic science front, further research into PEComa signaling networks and the tumor microenvironment (including tumor mutational burden and PD-L1 expression) may uncover new therapeutic targets and predictive biomarkers. Ultimately, prospective clinical trials of combination regimens and development of international consensus guidelines will be essential to improve outcomes for this rare disease.

## Conclusion

Malignant renal PEComa remains an exceptionally rare entity, especially in the setting of TSC. This case highlights several key points. First, although renal AMLs are common in TSC, malignant transformation into PEComa is exceedingly rare, particularly in young patients. This emphasizes the importance of vigilant surveillance (e.g. serial bilateral renal imaging) for large or atypical renal masses in TSC families. Second, definitive diagnosis requires tissue pathology; imaging alone cannot reliably distinguish malignant PEComa from benign AML or renal cell carcinoma. The presence of epithelioid morphology, melanocytic marker positivity, necrosis, or large tumor size should prompt consideration of PEComa in the differential diagnosis, especially in TSC patients. Third, prognostic factors such as size >5 cm, necrosis, high mitotic rate, infiltrative margins, elevated Ki-67, and TFE3 rearrangement should be integrated when assessing malignancy risk. In our patient, despite a relatively low Ki-67, the very large tumor size and necrosis warranted classification as malignant. Finally, advances in molecular oncology have confirmed the central role of mTOR dysregulation in TSC-related PEComa. mTOR inhibitors—especially albumin-bound sirolimus—are now the most effective systemic therapy for unresectable or metastatic disease. For localized tumors, surgical resection remains the mainstay, but targeted therapy has expanded treatment options for high-risk cases.

During outpatient consultations, such patients are typically initially seen in the dermatology department, whereupon a urology consultation should be arranged, or the patient should be referred to the urology department.

The presence of a family history and multi-system involvement (skin and kidney) in this case is typical of TSC-associated PEComa. However, the absence of neurological involvement in our patient, despite her brother’s severe epilepsy, highlights the significant intrafamilial phenotypic variability often seen in TSC2 mutations. This clinical heterogeneity can sometimes lead to a delayed diagnosis of malignant potential, as the focus may remain on managing benign AMLs. Our case demonstrates that even in the absence of debilitating symptoms like epilepsy, the high tumor burden (size >12cm) and rapid postoperative changes (the suspected recurrence at one month) necessitate aggressive management with mTOR inhibitors.

We acknowledge that the current report is limited by a short follow-up duration. Since the patient underwent surgery recently, long-term oncological outcomes regarding recurrence or distant metastasis remain to be determined. The discovery of a suspected enhancing mass at the one-month mark highlights the aggressive potential of malignant PEComa and the necessity for high-frequency imaging surveillance in the first postoperative year.

In summary, this case broadens the clinical spectrum of malignant renal PEComa and underscores the need for long-term follow-up, genetic evaluation, and consideration of mTOR-targeted strategies for optimal management. Given the paucity of data, further multicenter studies and molecular analyses are critically needed to improve diagnostic accuracy and therapeutic outcomes.

## Data Availability

The datasets presented in this study can be found in online repositories. The names of the repository/repositories and accession number(s) can be found in the article/supplementary material.
